# Early detection of ovarian cancer

**DOI:** 10.1007/s00330-020-06937-z

**Published:** 2020-05-28

**Authors:** Rosemarie Forstner

**Affiliations:** Department of Radiology, Universitätsklinikum Salzburg, Paracelsus Medical University, Müllner-Hauptstr. 48, A-5020 Salzburg, Austria

**Keywords:** Ovarian cancer, Epithelial ovarian cancer, Magnetic resonance imaging, Screening, Radiomics

## Abstract

**Abstract:**

Early detection is the only way to achieve a high cure rate in women with ovarian cancer. Unfortunately, to date, there is no effective strategy for early detection, despite rapidly emerging biomarkers. The low prevalence of ovarian cancer, low specificity and high rates of false positives have been limitations of screening programs. In the hands of experts, transvaginal sonography and MRI are effective tools to characterise ovarian masses. Currently, ongoing efforts in standardization of technique and analysis are likely to improve diagnostic capabilities in clinical routine, as well as the introduction of predictive risk models of malignancy. Radiomics and radiogenomics potentially offer a broad spectrum of complementary information in ovarian cancer diagnosis and treatment.

**Key Points:**

*• Transvaginal sonography and MRI are effective tools to characterise ovarian masses.*

*• Standardisation of imaging technique and implementation of predictive models of risk of malignancy contribute to early detection of ovarian cancer.*

From a clinical perspective, ovarian cancer remains a major challenge. Despite advances in therapy, only a marginal improvement in overall survival has been seen in the last decades. This is mainly attributed to the fact that ovarian cancer is mostly diagnosed late and subsequently will relapse. In contrast, borderline tumours and stage I invasive ovarian cancer have excellent prognoses. Unfortunately, early detection of ovarian cancer still remains one of the unmet needs in the management of this disease.

## Is an improved diagnostic pathway already in sight?

Undoubtedly the concept of ovarian cancer has been completely revised. Ovarian cancer is now recognised as an umbrella term for different cancer types that differ widely not only on a morphological and genetic level but also in clinical behaviour. Furthermore, heterogeneity is a feature seen not only within the primary tumour but also among its metastases [1].

**Fig. 1:**
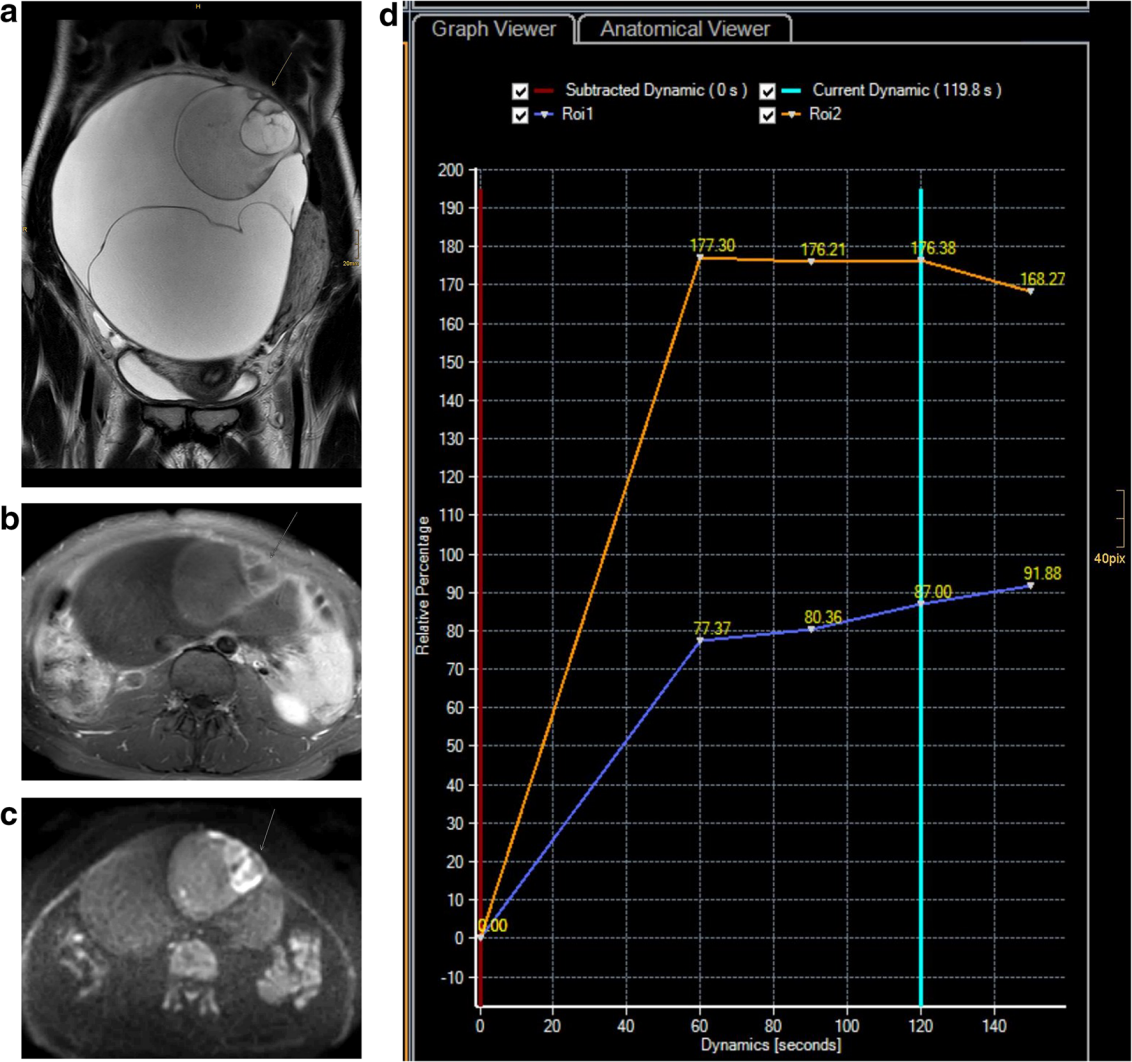
Mucinous borderline tumour and stage IA invasive ovarian cancer in a 28-year-old female. Coronal T2 (**a**) demonstrates a large multilocular cystic mass of the right ovary typical of a mucinous tumour. At its superior aspect areas with irregular septations, contrast enhancement (**b**) and restricted diffusion (**c**) are demonstrated (arrow). Time intensity curves of the uterus (orange) and solid tissue of the mass (blue) demonstrate type 2 curve with typical initial rise followed by a plateau (**d**). At histopathology, in this area, foci of invasive cancer were seen

Approximately 90% of ovarian cancers constitute of epithelial ovarian cancer types. Ovarian cancer has multiple cellular origins. The most common and aggressive type is high-grade serous ovarian cancer (HGSOC) which originates in the epithelium of the fallopian tube as a STIC lesion. HGSOC may manifest as an ovarian or fallopian tube mass or primary peritoneal cancer, and the term tubo-ovarian cancer is often used. In contrast, only the biologically more indolent type I cancers (low-grade serous, mucinous, endometrioid, and clear cell) derive from the ovaries. These two distinct cancer categories differ not only in origin and aggressiveness, but also in the presence of identified precursor lesions. In this context—although they comprise only the minority of ovarian cancers—it is pivotal for early detection that precursor lesions may precede ovarian cancer for several years. Serous and mucinous borderline tumours may arise within cystadenomas, and it seems that there is a continuum in their development to invasive cancers. This is supported by the fact that borderline tumours are diagnosed in women approximately 10 years younger than the average age for HGSOC and the coexistence of borderline and invasive cancer in the same histopathologic specimen (Fig. 1). Endometriosis is associated with clear cell and endometrial cancer subtypes.

Type I cancers tend to grow slowly and are likely to be diagnosed early by imaging. Unfortunately, for the vast majority of HGSOC, early detection is more challenging, as they disseminate early in the course of disease, within a few months, as evidenced by the screening studies in high-risk women. This may also be one of the reasons why the high expectations for screen detection of early ovarian cancer could not be fulfilled. But here we have to differentiate between women at high and those at low risk of ovarian cancer.

Genetic predisposition is associated with a higher risk of ovarian cancer that also tends to manifest at a younger age. BRCA1 and 2 mutation carriers harbour markedly increased life-time risk of ovarian cancer (40–45% resp. 15–20%) by the age of 70. The risk is low in high-risk women prior to the age of 40. This makes screening with transvaginal sonography (TVUS) an important tool for early diagnosis in this population and is the rationale for recommendations of semi-annual screening and risk reducing salpingo-oophorectomy as an efficient means for cancer reduction at ages of 35–40 years or after completion of childbearing [2].

Why do guidelines recommend against screening for women at normal cancer risk? The data from large randomised screening trials do not support benefits of screening outweighing the harms related to false positive testing. Furthermore, the rate of detected ovarian cancer is low, the performance in detecting stage I disease is limited, and survival benefit was not evident in comparison with not-screened women [2].

In the PLCO (prostate, lung, colorectal ovarian) cancer screening trial, no difference was found in the stage at diagnosis and the ovarian cancer death rate, but approximately 10% of participants had false positive results [2]. This resulted in a reported ratio of surgeries to cancer of approximately 20:1, and considerable complication rates were reported after surgery.

In the UKCTOCS trial with more than 50,000 postmenopausal women enrolled for annual TVUS, only 45 cancers were detected, and mortality reduction was not found in the screened women over a follow-up of 11 years. While concurrent Ca125 and TVUS screening was not effective, complementary TVUS performed in abnormal Ca-125 allowed diagnosing more early-stage cancers and borderline tumours [2].

So early detection of ovarian cancer must overcome problems of false positives derived from screening tests or pelvic ultrasonography and allow detection of preclinical ovarian cancer and precursors. The International Ovarian Tumour Analysis (IOTA) group published various models to standardise analysis of TVUS. While approximately 25% of adnexal masses remain sonographically indeterminate even with sonographic expert level, in clinical practice, this rate will be substantially higher [3]. Of note, these indeterminate masses mostly include benign masses such as benign teratomas, endometriomas, fibromas, or thecomas.

The complementary value of MRI lies in its ability to accurately characterise sonographic indeterminate masses. In an attempt to standardise ovarian mass assessment, the European Society of Radiology proposed an algorithmic pathway that allows for a specific diagnosis of most lesions and also serves as a guide for patient management [3]. A 5-point score has been proposed which includes the assessment of the perfusion of solid tissue using a time intensity curve, with the myometrium being used as the internal reference [4]. Recently, the O-RADS MRI score has been validated in a European multicentre study enrolling 1340 women [4]. The results confirm a robust score with sensitivities of 93% and specificities of 91% for detecting malignant lesions in sonographically indeterminate masses regardless of the level of radiological expertise. Its strength is underlined by the excellent positive likelihood for malignant masses (score 5). Of the 10% of masses scored as 4 (indeterminate), the proportion of malignant and benign pathologies was equivalent. Among these, borderline tumours were most commonly found (18.5%), while their rate was lower in scores 3 and 5. Data from this study provided the evidence for O-RADS MRI risk stratification scoring system that is aimed at a global standardisation of risk stratification and subsequently development of guidelines for management and follow-up of ovarian/adnexal masses using MRI, in conjunction with the O-RADS ultrasound score.

## Clinical implication of early detection of ovarian cancer

The ability to detect ovarian cancer before it metastasises is crucial, as borderline tumours and most stage I invasive cancers have a 5-year survival rate of more than 90%. Furthermore, accurate preoperative assessment may allow a tailored patient-centred approach for adnexal masses when these are assessed by US or in indeterminate cases complemented by MRI [2, 4]. Preoperatively identifying lesions as benign will prevent unnecessary surgery or allow minimally invasive approaches and reduce patient anxiety, whereas in suspected malignancy or indeterminate findings, referral to specialised cancer centres will guarantee adequate treatment. In young women presenting with borderline tumours, with early-stage epithelial or malignant germ cell cancers, fertility-preserving surgery and oocyte cryoconservation may be offered [4]. As many borderline tumours are diagnosed in fertile age, accurate preoperative assessment is crucial for patient counselling.

## Current research and perspectives

To date, no epigenetic biomarkers are available for the early detection of ovarian cancer from tissues or fluids. However, research detecting cancer at a preclinical stage is driven by rapidly advancing techniques. Gene expression– and methylation-based arrays and other emerging techniques such as liquid biopsies or autoantibody serum biomarkers are under development and have yet to be validated for early cancer detection.

The molecular classification of high-grade ovarian cancer (CLOVAR) allows distinction of four cancer subtypes that differ in genetic profiles and prognosis. Special imaging traits of peritoneal dissemination at the staging CT could be associated with these subtypes. Correlation of this subclassification using CT or MRI morphologic features or texture analysis rendered important prognostic information.

Radiomics and radiogenomics from CT or MRI data have opened new insights in ovarian cancer tumour biology. New, rapidly evolving applications include identification of radiomic features and their correlation with phenotype, genetic features, and prediction of cancer progression or prognosis [1]. The great advantage of radiomics in highly heterogeneous tumours such as HGSOC is the evaluation of the whole tumour/tumour burden which is, in contrary, not possible at a biopsy level. In this setting, a radiomic approach may allow the characterisation and quantification of inter- and intratumoural heterogeneity linked with prognosis and drug resistance [1]. Studies have shown, for example, dissimilarities in texture metrics between implants to be associated with poorer prognosis. Moving forward, the combination of radiomic features and clinical data may allow the creation of predictive models of resectability or of tumour progression [5]. However, before radiomics is integrated as a clinical adjunct, some hurdles (reproducibility, lack of automation,…) have to be overcome. Integration of artificial intelligence techniques will not only assist in solving these, but likely provide new prognostic algorithms for patient-tailored therapies.
